# Reduced Glutamatergic Currents and Dendritic Branching of Layer 5 Pyramidal Cells Contribute to Medial Prefrontal Cortex Deactivation in a Rat Model of Neuropathic Pain

**DOI:** 10.3389/fncel.2016.00133

**Published:** 2016-05-24

**Authors:** Crystle J. Kelly, Mei Huang, Herbert Meltzer, Marco Martina

**Affiliations:** ^1^Department of Physiology, Northwestern University Feinberg School of MedicineChicago, IL, USA; ^2^Department of Psychiatry and Behavioral Science, Northwestern University Feinberg School of MedicineChicago, IL, USA

**Keywords:** brain, chronic pain, EPSC, SNI, glutamate, dendrite, microdialysis

## Abstract

Multiple studies have demonstrated that neuropathic pain is associated with major reorganization in multiple brain areas. In line with the strong emotional salience of chronic pain, involvement of the limbic system appears particularly important. Within the past few years, it has become clear that the functional deactivation of the prefrontal cortex (PFC) is critical for both the cognitive/emotional and the sensory components of pain. However, at the cellular level, details of this deactivation remain in large part unclear. Here we show that 1 week after a peripheral neuropathic injury (Spared Nerve Injury model) pyramidal cells in layer 5 (L5) of the rat medial PFC show responses to excitatory glutamatergic inputs that are reduced by about 50%, as well as reduced frequency of spontaneous excitatory synaptic currents. Additionally, these cells have reduced membrane capacitance and increased input resistance. All these findings are consistent with decreased dendritic length, thus we performed a detailed morphological analysis on a subset of the recorded neurons. We found that the apical dendrites proximal to the soma (excluding the tuft) are shorter and less complex in SNI animals, in agreement with the reduced capacitance and glutamatergic input. Finally, we used *in vivo* microdialysis to compare the basal concentrations of glutamate and GABA in the PFC of sham and SNI rats and found that ambient glutamate is decreased in SNI rats. Taken together, these data show that impaired glutamatergic transmission contributes to the functional deactivation of the mPFC in neuropathic pain. Additionally, the reduced branching of apical dendrites of L5 pyramidal neurons may underlay the gray matter reduction in chronic pain.

## Introduction

Why does pain sometimes become chronic? Unfortunately, the mechanisms of pain chronification remain poorly understood, which is a major reason why no scientifically validated therapies exist for chronic pain. Studies performed in the past decade have shown that, in addition to DRGs and the spinal cord, supraspinal areas are also active participants in the mechanisms of pain chronification, and they can influence nociceptive processing at the level of the spinal cord (Jasmin et al., 2003; Johansen and Fields, 2004; Li and Neugebauer, 2004; Senapati et al., 2005; Tang et al., 2005; Wu et al., 2005; Li et al., 2010). In keeping with these results, several articles have shown that neuropathic pain is associated with functional and morphological changes in multiple brain areas, including a major involvement of limbic circuitry (Zhao et al., 2006; Metz et al., 2009; Li et al., 2010; Mutso et al., 2012; Chang et al., 2014) in line with the strong emotional salience of chronic pain (Wiech and Tracey, 2009; Darnall et al., 2014). Functional and morphological alterations of the prefrontal cortex (PFC) are found in different types of chronic pain including neuropathic (Metz et al., 2009; Cordeiro Matos et al., 2015) and arthritic pain (Ji et al., 2010), and involve the whole PFC, from the infralimbic region to the anterior cingulate cortex (ACC), although the polarity of the observed changes may vary in different regions or between cortical layers (Xu et al., 2008; Metz et al., 2009; Li et al., 2010; Santello and Nevian, 2015; Wang et al., 2015). A critical role of the PFC in chronic pain is further supported by the demonstration that a stronger initial functional connectivity between the PFC and the nucleus accumbens (NAc) predicts pain chronification (Baliki et al., 2012), thus suggesting that cortical activity is causal in the transition to chronic pain. Additionally, evidence of global reorganization of the mPFC in chronic pain derives form genome-wide methylation studies showing correlation between the methylation state of the PFC and pain tactile allodynia in SNI rats (Massart et al., 2016). At the circuitry level, it has been proposed that in chronic pain the PFC undergoes global deactivation, which then mediates the pain-associated cognitive impairments (Ji et al., 2010). Very recently, new indisputable evidence has appeared linking PFC deactivation with both the emotional and the sensory components of pain. Lee et al. (2015) have shown that acute optogenetic activation of the layer 5 (L5) pyramidal neurons of the prelimbic PFC relieves both the aversive cognitive symptoms of neuropathic pain as well as tactile and thermal allodynia. Similarly, Zhang et al. (2015) have shown that in the same cortical region, increased GABAergic activity from parvalbumin-positive interneurons leads to the inhibition of pyramidal cell firing. Intriguingly, the latter study also showed that optogenetic activation of parvalbumin-positive interneurons worsened the pain response, while their inhibition alleviated the pain phenotype. Thus, multiple lines of evidence concur that the PFC is deactivated in neuropathic pain and that this deactivation has a causal role in the phenotype. However, the cellular and molecular mechanisms of the PFC neurons remain still largely unexplored.

In this study, we take advantage of *ex-vivo* patch clamp recordings, morphological analysis of recorded pyramidal neurons and *in vivo* microdialysis to examine the cellular and molecular mechanisms affecting dorsal medial PFC (mPFC) activity in the spared nerve injury (SNI) model of neuropathic pain. All these different approaches support the idea of a general reduction in glutamate signaling in SNI animals. Additionally, morphological reconstructions of L5 pyramidal cells show evidence of a reduced dendritic arbor in SNI, which may contribute to the cortical gray matter reduction seen in neuropathic pain.

## Materials and Methods

### Ethics Statement

All animal procedures were performed with approval of the Northwestern University Institutional Animal Care and Use Committee.

#### Animals

Male, Sprague-Dawley rats were obtained from Charles River, housed in conventional rodent housing with unlimited access to food and water.

##### SNI Surgery

Spared nerve injury surgery was performed as described previously (Decosterd and Woolf, 2000). Briefly, isoflurane anesthesia was induced at 3l/min and then kept on a nose cone at 2.5–3l/min for the duration of the surgery. The sciatic nerve was exposed distal to the sural bifurcation. The tibial and peroneal nerves were tightly ligated with 6–0 braided silk sutures (Henry Schein) approximately 2 mm apart, and a 1–2 mm piece of nerve was removed between the ligations. The skin was then sutured with 4–0 nylon sutures (Henry Schein), triple antibiotic ointment was applied to the wound, and the animal was removed from anesthesia. For sham surgeries the nerve was exposed but it was not further manipulated, and the wound was immediately closed. All experiments were performed on young adult animals, 1 week after SNI (or sham) surgery. Ages of animals at the time of measurement were: p 44–46 for the microdialysis study; p 56–60 for spontaneous current recordings; and p 28–35 for recordings of evoked glutamatergic currents and morphological analysis.

##### Behavioral Assessment

Behavioral testing to determine the animals’ pain threshold was performed immediately prior to surgery and again 1 week post-surgery, prior to electrophysiological recording or one to 3 days after microdialysis. Rats were placed in a chamber with a metal mesh floor and acclimated for at least 20 min prior to testing. Testing was performed using von Frey filaments (Stoelting) of different strengths, which were applied to the plantar surface of the hind paws to determine the pain threshold. The 50% withdrawal threshold was calculated using the method previously described (Chaplan et al., 1994).

#### *In Vivo* Microdialysis

Male SD rats were used for these experiments. They were housed (maximum of 4/cage) in a controlled 14:10-h light-dark cycle with free access to food and water. Rats were randomly assigned to 2 groups: sham and SNI, (6 rats per group). At the age of p35–37 the following procedures were started:

##### Cannulation

At Procedural day 1 (p35–37), rats were anesthetized with 3% isoflurane. Guide cannulae (21 G) with dummy probes were then placed and fixed by cranioplastic cement to the mPFC. The stereotaxic coordinates of the implanted probes were the following: A +3.0, L −0.8 (10° inclination), V −4.0 mm.

Two days after cannulation (Procedural day 3), SNI or sham surgery were performed as described above.

##### Dialysis

At Procedural day 9, concentric-shaped dialysis probes (Synaptech Co.), with 2 mm non-glued membrane surface were implanted into the rats’ mPFC under light isoflurane anesthesia. Probes were implanted in the morning, with perfusion speed set at 0.8 μl/min. The perfusion medium was Dulbecco’s phosphate-buffered saline solution (Sigma), containing (in mM): 138 NaCl; 8.1 Na_2_HPO_4_; 2.7 KCl; 1.5 KH_2_PO_4_; 0.5 MgCl_2_; 1.2 CaCl_2_, pH = 7.4). The flow rate was reduced to 0.2 μl/min during overnight perfusion. In the morning of the next day, the flow rate was again increased to 0.8 μl/min, and four dialysate samples were collected at 30 min intervals. For each animal, the average baseline glutamate and GABA levels were calculated by averaging the values obtained from the four, 30 min samples. This average was then used as a single data point for each of the 12 animals (6 sham and 6 SNI). After the dialysis was completed, the animals were tested for allodynia as described above and sacrificed to confirm the location of the probes by manual brain dissection (100 μm slices).

##### Dialysis Samples Storage and Quantification

Samples collected 7 days after sham/SNI surgery were stored at −80°C until used. Neurotransmitter concentrations were assayed by Ultra-Performance Liquid Chromatography (UPLC) and Tandem mass spectrometry, as described in a recent publication (Huang et al., 2014). The LC system (Waters Acquity UPLC, Waters Co.) with a binary solvent manager, sample organizer, sample manager and column manager and a Waters Acquity UPLC HSS T3 1.8 μm column were used for the separation. The UPLC system was coupled to a triple-quadrupole mass spectrometer (Thermo TSQ Quantum Ultra), using ESI in positive mode. All data were processed by Waters MassLynx 4.0 and Thermo Xcaliber Software. Data were acquired and analyzed using Thermo LCquan 2.5 Software (Thermo Fisher Scientific).

#### *Ex-vivo* Electrophysiological Recordings and Morphological Analysis

##### Slices and Solutions

7–11 days after SNI/sham surgery, rats were anesthetized with a lethal dose of Ketamine/Xylazine (3:1 ratio) and perfused with ice-cold, oxygenated, ACSF. Rats were then decapitated and the brain removed into ACSF slush. Acute slices were obtained using a Microm Vibratome (Thermo-Scientific). Three hundred micrometers coronal sections containing medial prefrontal cortex (mPFC) were obtained, stored for 20 min at 32–35°C, brought to room temperature, and stored up to 8 h until use. For spontaneous EPSC recordings, perfusion, slicing and storage were performed using low-Ca^2+^, high-Mg^2+^ artificial cerebral spinal fluid (ACSF) containing (in mM): 125 NaCl, 25 NaHCO_3_, 2.5 KCl, 1.25 NaH_2_PO_3_, 25 glucose, 0.5 CaCl_2_ and 7 MgCl_2_. For recordings of evoked currents, perfusion, slicing and storage were performed using N-methyl-D-glucamine (NMDG)-substituted ACSF containing (in mM): 92 NMDG, 2.5 KCl, 1.25 NaH_2_PO_3_, 30 NaHCO_3_, 20 HEPES, 25 glucose, 2 thiourea, 5 Na-ascorbate, 3 Na-pyruvate, 0.5 CaCl_2_ and 10 MgCl_2_, pH 7.4 with HCl. For all electrophysiological recordings, slices were transferred one at a time into a recording chamber containing standard ACSF (in mM): 125 NaCl, 25 NaHCO_3_, 2.5 KCl, 1.25 NaH_2_PO_3_, 25 glucose, 2 CaCl_2_ and 1 MgCl_2_. Spontaneous currents were recorded at 30–32°C. Evoked currents were recorded at room temperature. Slices were exposed to a continuous flow of oxygenated ACSF (bubbled with 95% O_2_ and 5% CO_2_). For spontaneous EPSC recordings, pipette internal solution contained (in mM): 138 Cs-methanesulfonate, 10 EGTA, 10 HEPES, 2 MgCl_2_, 2 NaCl, 56.6 Sucrose, 5 QX-314 and 1 mg/ml biocytin, pH 7.3 with CsOH. For evoked current recordings, pipette internal solution contained (in mM): 138 K-gluconate, 2 MgCl_2_, 2 Na_2_ATP, 0.2 NaGTP, 0.1 EGTA, 10 HEPES, 2 NaCl, 5 QX-314 and 1 mg/ml biocytin, pH 7.3 with KOH. Drugs used in the experiments were applied through the bath solution.

##### Electrophysiological Recordings

Slices were obtained from SNI- and sham-operated rats 7–11 days post-surgery. The right hemisphere (contralateral to surgery) dorsal region of mPFC was targeted for patch clamp recordings. Pyramidal neurons in L5—between 500 and 1000 μm from the pial surface—were selected using DIC microscopy based on their large cell bodies and visible apical dendrites. Cell location was recorded using a calibrated manipulator (Luigs and Neumann) for the microscope stage. Patched cells were filled with biocytin (1 mg/ml) for *post hoc* morphological reconstruction. Cells were patched in whole-cell configuration using pipettes made from 1.5 mm thick-walled borosilicate glass (Sutter) pulled to a tip size of 5–10 MΩ with a Sutter horizontal puller. Electrophysiological data were obtained using an Axopatch 200B amplifier and acquired and processed using pClamp9 Software. EPSC recordings were performed at a holding potential of −70 mV. Spontaneous EPSC recordings of 100 s duration were analyzed using custom MATLAB Software. Twenty milliMolar CNQX was bath applied to a number of cells to confirm that the spontaneous currents were AMPA-mediated. Synaptic currents were evoked using a bipolar electrode placed in layer 2/3. The responses to extracellular stimulations (delivered in 200 μA increments) were measured until the evoked current remained in a linear range. For each cell, to measure response magnitude, the maximum current and the slope of the linear range of responses were calculated. Passive cell membrane properties were calculated from hyperpolarizing current injections (−50 pA steps) in current clamp. Membrane input resistance (Rm) was calculated from the peak voltage response to a current injection of −100 pA. The contribution of the hyperpolarization-activated current (Ih) was estimated from the voltage responses to hyperpolarizing current injections that produced a peak value nearest −105 mV and calculated the percent difference between peak voltage (V_PEAK_) and the steady state voltage (V_SS_). Membrane capacitance (Cm) was obtained from the pClamp membrane test function. Specific Rm (Rm × cell surface area) was calculated for all cells for which a morphological reconstruction was performed (see below). For these data, Rm was calculated in voltage clamp using the pClamp membrane test function. Cell surface area was estimated assuming a constant dendritic diameter of 1 μm. Comparing the absolute size of currents elicited by tract stimulation in different slices may be problematic because the current amplitude can be affected by multiple variables including small differences in the relative positions of the recording and stimulating electrode, overall slice quality, and small variations in bath temperature and flow. Thus, in addition to the size of the maximum amplitude we also investigated the slope of the increase in evoked response magnitude with increasing stimulation intensity (I/O slope), a measure that is more robust and reproducible between slices. All electrophysiological analysis was performed in blinded conditions.

##### Morphological Reconstruction and Analysis

Immediately following electrophysiological recording, slices were moved to a 4% paraformaldehyde solution in PBS and fixed. Slices were then transferred to PBS and washed 3× for 15 min on a rotating platform. Endogenous peroxidases were quenched by incubating 30 min in PBS containing 1% H_2_O_2_ and 10% methanol. After another three rinses in PBS, tissue was permeabilized for 1 h in 2% Triton X-100 (Sigma) in PBS. Slices were then incubated for 2 h with ABC reagent (ABC vectastain kit from Vector Labs) using PBS with 1% Triton X-100 as the buffer. Slices were rinsed thoroughly 3 × 15 min in PBS followed by one rinse for at least an hour. To visualize staining, slices were incubated in DAB (Sigma) made from tablets dissolved just prior to use. Slices were monitored until staining was sufficient, then removed to PBS and rinsed twice to stop the DAB reaction. Slices were mounted using the aqueous anti-fade mounting medium Mowiol (Sigma). Well-filled cells were selected and traced by hand under blind conditions using a Zeiss Axioscop 2 microscope and drawing tube. Traced images were then scanned into a personal computer and reconstructed using Neuromantic Software (freely available from the site of the University of Reading, UK; http://www.reading.ac.uk/neuromantic/body_index.php). The resulting reconstruction was analyzed for dendritic length and branching of the total dendritic tree or for the basal, apical, apical tuft or proximal apical (apical dendrite excluding the tuft) dendritic arbors separately.

### Statistics

**Figure 1:**
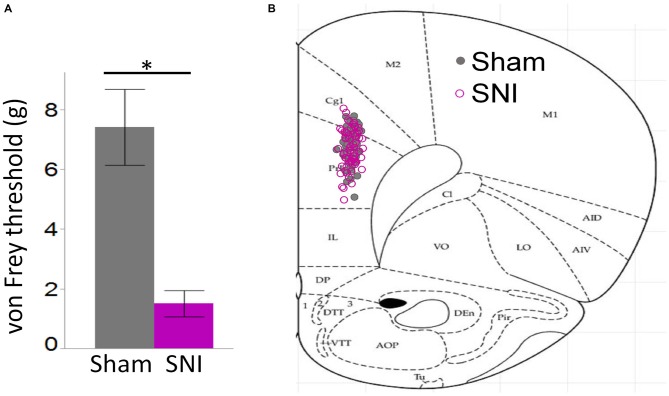
**Behavioral pain assessment of rats and location of neurons utilized in this study. (A)** Von Frey measurements in the injured paw revealed a strong tactile allodynia in SNI animals compared to sham (32 sham and 30 SNI rats; *p < 0.001, Student’s *t*-test, two-tailed). **(B)** Locations of 61 sham and 62 SNI Layer 5 (L5) pyramidal neurons that were used for electrophysiological characterization in sham and SNI rats.

**Figure 2:**
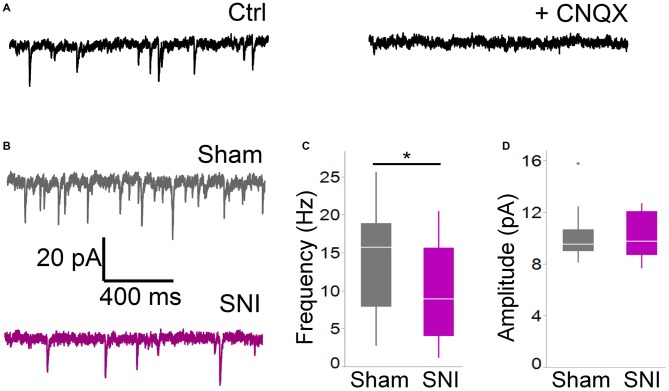
**Spontaneous glutamatergic currents are reduced in SNI animals. (A)** Spontaneous EPSCs recorded from mPFC L5 pyramidal cells at −70 mV in cells are blocked by the AMPA channel blocker CNQX (20 μM). The traces shown here were obtained in a slice from a sham animal. **(B)** Spontaneous EPSCs were recorded 1 week after surgery in slices from sham (top trace) and SNI (bottom trace) animals. **(C,D)** sEPSCs were reduced in frequency (**C**; 21 cells from sham and19 from SNI slices; *p = 0.045, Wilcoxon rank-sum test, two-tailed), but not in amplitude **(D)**. Recordings were performed at 30 ± 1°C.

**Figure 3:**
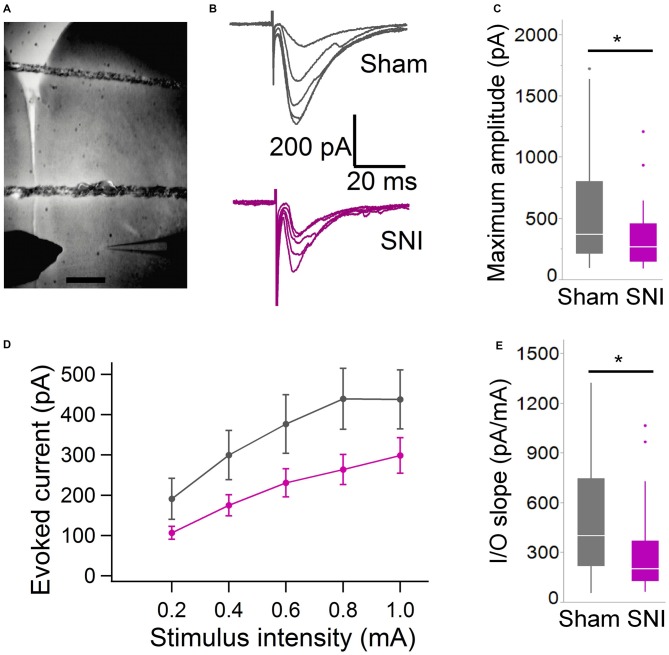
**Evoked glutamatergic currents are smaller in SNI animals. (A)** Microphotograph showing the placement of bipolar stimulation electrode in L2 adjacent to recording electrode in L5. Scale bar = 300 μM. **(B)** Current traces recorded (at 22 ± 2°C) from individual cells from sham (top) and SNI rats (bottom traces) in response to extracellular stimulation of increasing magnitude (from topmost trace, 200, 400, 600, 800 and 1000 μA stimuli.) **(C)** The maximum evoked current was significantly smaller in SNI compared to sham (*p = 0.025, Wilcoxon rank-sum test, two-tailed). Stimulus intensities used to evoke the maximal current were not different between sham and SNI (not shown). **(D)** Plot showing the input-output responses across a range of stimulus intensities from 200 to 1000 μA. **(E)** The slope of the I/O function was reduced in SNI cells. The input-output slope in the linear range was determined for each cell. The bar chart summarizes the data from 29 sham and 31 SNI cells (*p = 0.012, Wilcoxon rank-sum test, two-tailed).

**Figure 4:**
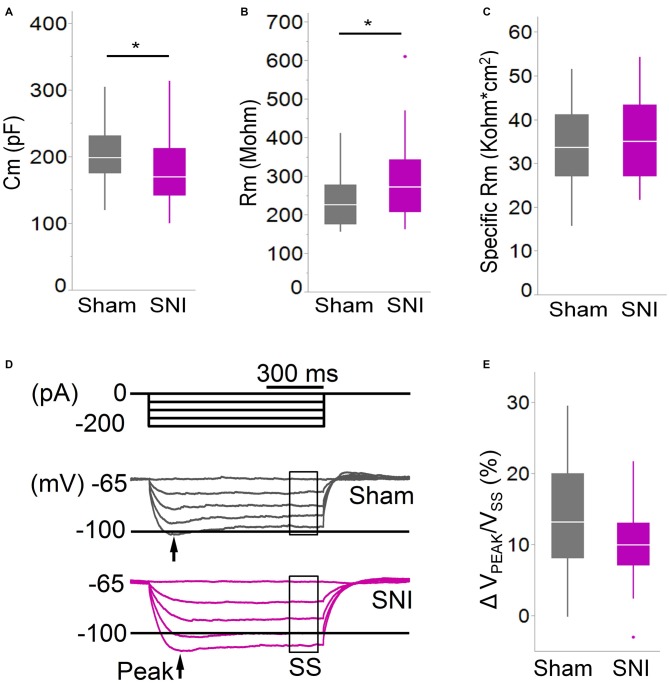
**Membrane properties of mPFC L5 pyramidal neurons are altered in SNI rats. (A)** The membrane capacitance (Cm) of L5 pyramidal cells was decreased (*p = 0.038, Wilcoxon rank-sum test, two-tailed), while **(B)** the membrane input resistance (Rm) was increased (*p = 0.048, Wilcoxon rank-sum test, two-tailed) in slices from SNI animals 1 week after surgery. However, the specific membrane resistance **(C)** was unaffected, suggesting that the change in resistance is driven by that in capacitance. **(D,E)** The voltage sag ratio in response to hyperpolarizing current injection was only slightly decreased in SNI (*p* = 0.153, Wilcoxon rank-sum test, two-tailed). Recordings were obtained at 22 ± 2°C.

**Figure 5:**
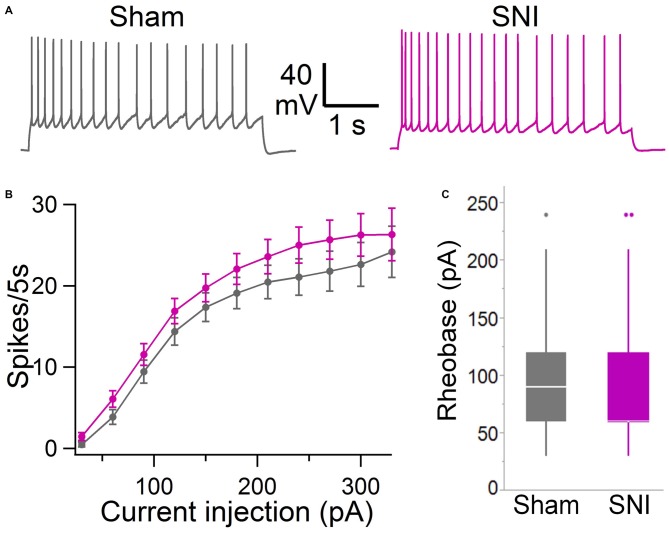
**Intrinsic excitability of mPFC L5 pyramidal neurons is not altered in SNI rats. (A)** Voltage responses to depolarizing current injection (150 pA, 5 s) obtained in sham (black trace) and SNI (purple trace) condition. Recordings were performed at 30 ± 1°C in the presence of picrotoxin (0.1 mM) and kynurenic acid (3 mM). **(B)** No significant difference was observed in the F/I curve (sham, 64 cells; SNI, 79 cells; two-way ANOVA, *F* = 0.15, DF = 1, *p* = 0.70). **(C)** Rheobase current was also not significantly different in slices from SNI compared with sham animals (89 ± 6 pA vs. 92 ± 6 pA; 79 and 64 cells, respectively; *p* = 0.22, Wilcoxon rank-sum test, two-tailed).

**Figure 6:**
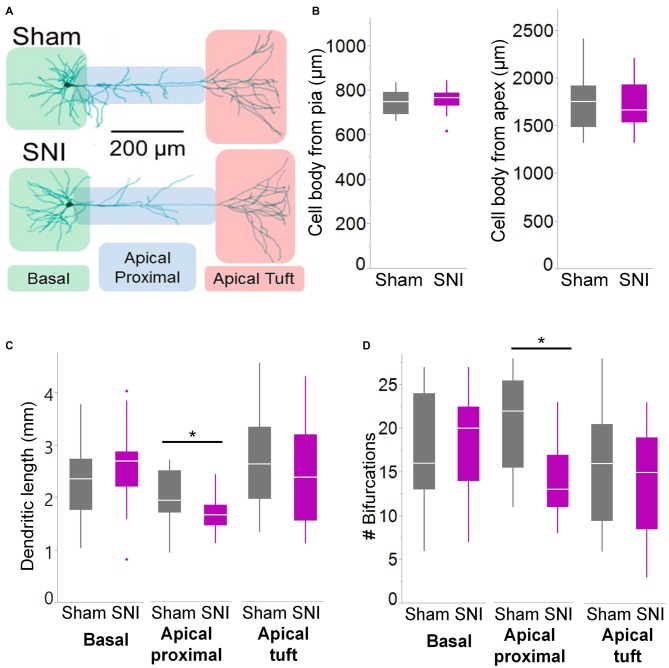
**Branching of apical dendrites is reduced in pyramidal cells from SNI animals. (A)** Biocytin-filled L5 mPFC pyramidal neurons from sham and SNI rats were traced by hand and reconstructed with Neuromantic Software. **(B)** The reconstructed cells from the two experimental groups had overlapping location. **(C,D)** Dendritic morphology was altered in neurons from SNI animals (*n* = 17 SNI, 17 sham), specifically in the proximal apical dendritic region (the apical dendrite excluding the tuft). This includes a reduction in total length (**C**, apical proximal dendrites *p = 0.018, Wilcoxon rank-sum test, two-tailed) and a reduction in dendritic branching (**D**, apical proximal dendrites *p = 0.003, Wilcoxon rank-sum test, two-tailed).

Behavioral and microdialysis data were analyzed using a Student’s *t*-test. Most of the electrophysiological and morphological data collected were not normally distributed; therefore a two-tailed Wilcoxon rank-sum test (also known as Wilcoxon-Mann-Whitney) was used to determine statistical differences between sham and SNI surgery groups. Differences were considered significant for *p*-values < 0.05. All results in the text and Figures 1, 6 are given as mean ± standard error of mean (SEM). In Figures 2–5 data are presented as box plots showing 75th and 25th quartiles. Whiskers extend to points that fall within the 75th quartile + 1.5 × (interquartile range) and 25th quartile − 1.5 × (interquartile range).

## Results

The prelimbic cortex output is deactivated in neuropathic pain. While evidence has been provided pointing at increased GABAergic inhibition from parvalbumin-positive cells as a contributor to the deactivation (Zhang et al., 2015), little is known about glutamatergic currents, which provides the main excitatory drive to the PFC. As L5 provides the main integrated output of this region, we focused our investigation on this cortical layer. As a first general approach, we performed patch clamp recordings from acute cortical slices to compare the strength of spontaneous synaptic glutamatergic currents in L5 pyramidal cells form pain and control animals 1 week after a peripheral neuropathic lesion.

For these experiments we took advantage of the SNI model of neuropathic pain to study glutamatergic inputs to the PFC of control and pain animals. One week following sham/SNI surgery, rats were behaviorally tested to assess their tactile thresholds. As expected, the pain threshold in the injured (left) paw of SNI animals was markedly decreased compared with sham-operated rats (1.5 ± 0.44 g in SNI, vs. 7.42 ± 1.27 in sham, 32 and 30 animals, respectively, *p* < 0.001, Figure 1A). No significant differences were observed between the uninjured paws (SNI, 11.28 ± 2.21 g vs. sham 9.95 ± 1.88 g; not shown). The rats were then sacrificed for *ex-vivo* electrophysiological recordings from acute PFC slices. All the cells patched for these studies were located in the dorsal region of the medial PFC (mPFC), at a distance between 971 and 3247 μm (mean distance: 1855 ± 36 μm) from the slice apex (Figure 1B). Recordings of spontaneous excitatory currents (EPSCs) were made in whole-cell configuration at −70 mV. The currents recorded in these conditions were mediated by AMPA glutamate receptors, and were blocked in the presence of 20 μM CNQX (Figure 2A). We found that the frequency of spontaneous EPSCs was almost halved in cells from SNI (9.8 ± 1.5, Hz, *n* = 19) compared to sham animals (14.5 ± 1.5 Hz, *n* = 21; *p* = 0.045; Figure 2C). However, no significant difference was detected in event amplitude (10.1 ± 0.4 pA in SNI vs. 10.1 ± 0.4 pA in sham; *p* = 0.935; Figure 2D).

To further characterize the glutamatergic responses in these cells, we studied the macroscopic currents elicited by electrical stimulation of the afferent inputs. To this end, cells were voltage clamped at −70 mV in whole-cell configuration and stimulated using a bipolar electrode placed in layer 2/3 (Figure 3A). The maximum evoked current was significantly smaller in SNI (332 ± 45 pA, *n* = 31) compared to sham (550 ± 82 pA, *n* = 29; *p* = 0.026; Figure 3C). Additionally, the slope of the linear range of the input-output relationship was also significantly smaller in SNI cells (302 ± 45 pA/mA) compared to sham (515 ± 72; *p* = 0.017; Figure 3E). Thus, spontaneous and evoked glutamatergic currents are reduced in SNI and therefore contribute to the overall cortical deactivation. Neuronal activity, however, is the compound effect of synaptic and intrinsic excitability. Thus, we next analyzed the cable properties of PFC pyramidal neurons in sham and SNI rats (Figure 4). We found that both the membrane capacitance (Cm) and the input resistance (Rn) differed between the two experimental groups. The input resistance was significantly larger in SNI compared to sham (292 ± 21 vs. 237 ± 15 MΩ; 26 and 21 cells respectively; *p* = 0.048; Figure 4B), while the membrane capacitance was reduced in SNI cells (183 ± 10 pF, *n* = 31 vs. sham, 205 ± 8 pF *n* = 29; *p* = 0.038; Figure 4A). Interestingly, in a subset of cells for which we reconstructed the whole dendritic arbor, the specific membrane resistance was similar in cells from SNI and sham rats (35.7 ± 2.3 vs. 34.7 ± 2.5 KΩ·cm^2^, *n* = 17 in each group; *p* = 0.757; Figure 4C), suggesting that the increased input resistance largely depends on the smaller capacitance. The small (non-significant) reduction in the voltage sag in response to hyperpolarizing current injection (the sag ratio was 12.0 ± 1.2 in 28 SNI recordings vs.; 15.2 ± 1.7 in 28 sham recordings; *p* = 0.153; Figures 4D,E) suggests that the smaller capacitance may be caused by smaller dendritic surface, because Ih density in these cells increases in distal dendrites (Stuart and Spruston, 1998; Berger et al., 2003). In line with the interpretation that changes in Rm and Cm reflect a reduction in length/number of apical dendritic branches without alterations of the intrinsic membrane properties, no significant difference in excitability (rheobase and F/I curve; Figure 5) of L5 neurons was detected between sham and SNI rats in response to somatic current injections in the presence of blockers of fast GABAergic and glutamatergic synaptic transmission, as previously reported by Zhang et al. (2015). Thus, the cable properties suggest that L5 pyramidal cells from SNI rats have shorter dendrites. To verify this hypothesis, we performed a *post hoc* morphological analysis on the cells for which we reconstructed the dendritic arbor. This analysis was performed on a subset of neurons (*n* = 17 each for SNI and sham) that were best filled (Figure 6). Similar to the larger population of cells used for the electrophysiological recordings, cells from SNI and sham rats in this subset also showed complete overlap with regard to position within the slices (Figure 6B). We found that cells from SNI animals show a selective decrease in the length (1.76 ± 0.08 mm in SNI vs. 2.14 ± 0.12 in sham; *p* = 0.018; Figure 6C) and number of branches (14.2 ± 1.0 vs. 20.4 ± 1.4; *p* = 0.003, Figure 6D) of the apical dendrites (localized to layers 2–3 and shallow L5), while no difference was detected in basal dendrites or the apical tuft. The decreased dendritic length is consistent with the differences in cable properties and in glutamatergic currents.

**Figure 7:**
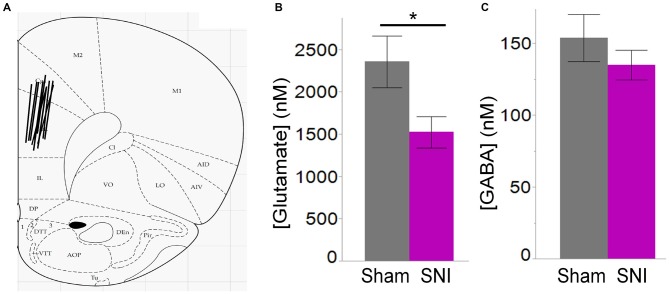
**Ambient glutamate is reduced in the mPFC of SNI rats. (A)** Location of the 2 mm probes used to collect ACSF dialysate in the dorsal region of the medial prefrontal cortex (PFC) in sham and SNI rats. The black lines represent the positioning of the 2 mm probes in each animal. **(B)** Glutamate concentration was significantly reduced in the mPFC 7 days post-SNI (*n* = 6 sham, 6 SNI; *p < 0.05, Student’s *t*-test, two-tailed). **(C)** GABA concentration was unaltered in the mPFC 7 days post-SNI.

Thus, electrophysiological and morphological data support the hypothesis that glutamatergic excitation is reduced in L5 pyramidal cells of SNI animals, due to diminished input, decreased macroscopic current, and reduced dendritic branching of the apical trunk. Finally, we wondered whether impaired glutamatergic drive could also be the consequence of altered release. To this end we used *in vivo* microdialysis to measure the average concentration of the main excitatory (glutamate) and inhibitory (GABA) neurotransmitters in the PFC of sham and SNI rats. As shown in Figure 7, we found that ambient glutamate concentration was reduced in the PFC of SNI rats 1 week after surgery (SNI: 1529 ± 184 nM, *n* = 6; sham: 2362 ± 305 nM, *n* = 6; *p* = 0.047; Figure 7B), while GABA concentration was unaltered (SNI: 135 ± 10 nM, *n* = 6; sham: 154 ± 16 nM *n* = 6; *p* = 0.356; Figure 7C). Thus, both pre- and postsynaptic factors appear to contribute to cortical deactivation as early as 1 week after a peripheral neuropathic injury.

## Discussion

### Glutamatergic Signaling is Impaired in SNI Animals

In this study, we show that impaired glutamatergic signaling contributes to the general deactivation of L5 PFC neurons 1 week following SNI surgery. This conclusion is supported by several experimental results: (1) Spontaneous EPSCs are reduced in frequency in L5 pyramidal neurons. (2) Electrically evoked EPSCs are diminished in this region. (3) The length and branching of apical dendrites (excluding the tuft) are reduced. Finally, (4) The microdialysis study shows that, at the same time point after surgery, glutamate concentration in the mPFC is significantly reduced in SNI animals. General mPFC deactivation was initially proposed by Ji et al. (2010) in a rat model of arthritis pain. More recently, two elegant articles employing optogenetic approaches have provided strong support to this idea. One of these articles (Lee et al., 2015) showed that selective optogenetic activation of the prelimbic cortex output to the NAc relieves both the emotional (anhedonia) and sensory pain components. The second article (Zhang et al., 2015) showed that increased inhibition (by optogenetic excitation using ChR2) from parvalbumin-positive interneurons in L5 of the prelimbic cortex worsens neuropathic pain, while decreased inhibition (by hyperpolarizing the interneurons using Arch3) alleviates pain. In line with this latter observation, it was newly shown that cannabinoid receptor mediated depolarization induced suppression of inhibition (DSI) is impaired in the infralimbic cortex of a rodent model of arthritic pain (Kiritoshi et al., 2016). Thus, the decreased glutamatergic excitability may also directly affect the GABAergic activity creating a reinforcing loop that leads to further cortical deactivation. In this context, it is worth noting that we recorded sEPSC in the absence of any GABA blockers. Thus, the decreased sEPSC frequency may be the consequence of the increased inhibition of neighboring and/or contralateral pyramidal cells (which provide commissural projections as well as collaterals) as well as of the possible increased GABA_B_ inhibition of glutamatergic terminals. It is also worth noting that our inability to detect changes in the basal GABA concentration in the mPFC of SNI animals cannot be viewed as contradiction of the alteration in GABAergic inhibition due to several reasons. First, increased inhibition was described in L5 of the prelimbic cortex and the probes that are used for microdialysis are large relative to the size of individual cortical layers or even PFC subdivisions. Second, we collected samples for 30 min; thus changes due to the typically fast and synchronized inhibition provided by parvalbumin positive cells (Fricker and Miles, 2001; Hefft and Jonas, 2005) would be diluted. Third, the ambient GABA concentration depends on GABA reuptake, which may also be altered in pain. Finally, the increased inhibition may at least in part be mediated by postsynaptic mechanisms, such as increased GABA channel density. Additionally, it is important that our finding that ambient GABA is unaltered in SNI rats 1 week after surgery is similar to that in CD1 mice 1 month after surgery (Guida et al., 2015). Further studies on the mechanisms of GABAergic inhibition in pain will help clarify these points that are crucial to understanding the mechanisms at the base of pain behavior which is critically dependent on PFC activity (Neugebauer et al., 2009; McDonald and Hong, 2013).

### Are the Observed Changes Input Specific?

One difficulty shared by any electrophysiological study of a complex area such as the PFC concerns the large cell-to-cell variability, which is due to the heterogeneity of pyramidal cell types in PFC. These cells differ in their morphological and functional properties and in their connectivity. The PFC receives sensory input from the thalamus as well as limbic input from hippocampus, amygdala and contralateral PFC (Hoover and Vertes, 2007). Thus it will be interesting to test whether the observed differences are circuit-specific. It is noteworthy that dendritic alterations appear specifically localized to dendritic segments located in layer 2/3 and shallow L5. It has been shown that inputs to layer 2 mPFC are non-uniform in their sub-cellular synaptic distribution (Little and Carter, 2012). Different inputs target different parts of the dendritic arbor, and this is likely true for L5 pyramidal neurons as well. Therefore, we suggest that alterations in the mPFC are likely due to changes in inputs that selectively synapse onto dendrites in layer 2/3 and shallow L5. The input from ventral hippocampus is a likely candidate due to the distribution of terminals which are concentrated in these layers (Dembrow et al., 2015). Because hippocampal function is impaired in SNI animals (Ren et al., 2011; Mutso et al., 2012) it can be expected that the strength of hippocampal inputs to the PFC is decreased in SNI animals. This hypothesis fits well a scenario where, in the pain condition, fear-related inputs from the amygdala become preponderant, while the hippocampus and PFC are deactivated. This maladaptive pattern is also observed in other disorders of sensory and emotional processing, such as PTSD (Bremner, 2006; Shin et al., 2006). It is also important to consider that alterations in membrane properties that are restricted to particular neuronal segments may importantly affect the neuronal functional map (Narayanan and Johnston, 2012) and thus further amplify the effects of potential alterations in circuit-specific inputs. Our results, however, suggest that the intrinsic membrane properties remain unaltered in SNI, and that the alterations in electrophysiological parameters are mainly the result of the morphological changes. Future experiments, possibly taking advantage of opto- or chemo-genetic strategies to isolate the hippocampal from other PFC inputs, will be necessary to directly test the hypothesis that hippocampal inputs to the PFC are selectively affected in SNI animals. The same type of experimental approach may be used to address another important point that still has no final answer: whether the pain-associated increase in inhibition is a widespread phenomenon or a cell-type and/or layer selective phenomenon. As discussed above, a possible interpretation of the microdialysis data may be that that the changes in inhibition are circuit-selective, but opto- or chemo-genetic analysis will be required to provide a final answer. However, while it is likely that many of the pain-associated modifications are circuit-specific, recent studies investigating the transcriptome and epigenetic markers in the PFC of pain animals suggest that this region undergoes a global reorganization that involves the expression of hundreds of genes (Alvarado et al., 2013; Massart et al., 2016).

## Author Contributions

CJK performed the electrophysiological recordings and morphological analysis; analyzed the data and co-wrote the manuscript. MH performed the microdialysis and analyzed the data; drafted the manuscript. HM designed the microdialysis experiments and provided critical reading of the manuscript. MM designed the project and all the experiments. Supervised data analysis and wrote the manuscript.

## Conflict of Interest Statement

The authors declare that the research was conducted in the absence of any commercial or financial relationships that could be construed as a potential conflict of interest.
